# Improved one-dimensional residual network high-voltage DC diagnosis for high-precision fault identification

**DOI:** 10.1371/journal.pone.0341115

**Published:** 2026-01-20

**Authors:** Rui Li, Xiaopeng Zhang, Wei Hao, Ting Wang

**Affiliations:** State Grid Shanxi Electric Power Research Institute, Taiyuan, China; Hanshan Normal University, CHINA

## Abstract

High-Voltage Direct Current (HVDC) transmission systems require fast and reliable fault diagnosis to ensure secure and stable operation. However, existing methods, including conventional Convolutional Neural Networks (CNNs), often suffer from limited accuracy and degraded training performance as network depth increases. To address these limitations, this study proposes an improved one-dimensional Residual Neural Network (1D-ResNet) that integrates an attention mechanism within the residual blocks to enhance feature extraction, stabilize gradient propagation, and accelerate model convergence. A comprehensive simulated HVDC platform is established to generate multiple fault scenarios, and the proposed network is trained to identify one normal condition and six typical fault types. Experimental results demonstrate that the proposed method achieves an average diagnostic accuracy of 99.15%, outperforming traditional CNN-based approaches by 12.89%. Moreover, the loss value is significantly lower than that of the conventional CNN model, indicating substantial improvements in both robustness and learning efficiency. These findings confirm the effectiveness of the proposed attention-enhanced residual framework for high-precision HVDC fault diagnosis.

## Introduction

### Background

Electric power occupies an irreplaceable and pivotal role in the national economy and daily life [1]. High-voltage direct current (HVDC) transmission technology has been widely adopted in recent years owing to the growing demand for long-distance, high-capacity power transmission, along with the increased requirements for grid stability and controllability. Furthermore, it has contributed to the rapid advancement of power electronics technology, and promotion of energy-saving and environmental protection initiatives [2]. When compared with conventional high-voltage alternating current (HVAC) transmission, HVDC exhibits lower energy losses, enhanced stability, and superior system control performance over long distances, thereby contributing significantly to modern power systems [3].

China’s Direct Current (DC) power transmission system is expanding rapidly, presenting an increased probability of faults [4]. Consequently, the research and development of relay protection technology tailored to the characteristics of DC systems has become essential for ensuring safe and stable operation [5]. Relay protection technology for high-voltage DC transmission lines primarily encompasses fault diagnosis, isolation, and recovery [6]. This enables the system to promptly and accurately protect transmission lines and the corresponding equipment in the event of a fault. The rapid development of artificial intelligence (AI) technology has enabled a more effective analysis of the fault characteristics in power systems owing to the powerful learning capabilities of AI [7]. The fault characteristics can be identified more accurately through AI-driven analysis, presenting a more reliable fault diagnosis and decision-making when compared with the conventional methods [8].

### Literature review

Furthermore, extensive research has been conducted on the application of AI technology in fault diagnosis for HVDC transmission systems both domestically and internationally [9]. Various machine learning techniques, including support vector machines (SVM), random forests (RF), artificial neural networks (ANN), and back-propagation neural networks (BP), have been proposed to address the fault diagnosis in HVDC transmission lines [10]. Feature extraction and classification are crucial components in power quality event classification [11].

Johnson et al. [12] employed SVMs to identify and classify faults in HVDC lines by extracting the standard deviation of the signals within half a cycle, before and after a fault, as the feature vector for classification. However, the fault tolerance of this method must be further validated. R.

Kou et al. [13] employed BP neural network training for the fault diagnosis of transmission lines using the electrical variation observed during the transmission line faults as the feature vector. However, this method cannot be used to identify faults that occur out-side the protected area, which introduces certain limitations.

K. Moloi et al. [14] employed intelligent optimization algorithms to refine a neural network. In particular, they adopted particle swarm optimization (PSO) to optimize an ANN, which was subsequently used to identify and classify various faults.

Wu et al. [15] employed RF neural networks to select the fault poles and identify fault types in HVDC transmission lines. However, the fault feature extraction process used in this method is relatively complex and exhibits certain uncertainties.

[16] proposed a fault detection approach for HVDC transmission lines that integrated a wavelet transform and an ANN optimized through ant colony optimization. This method selects the most representative features from the voltage, current, and their derivative signals, thereby achieving effective fault detection and classification.

[17] proposed a single-terminal fault detection method using a closed-form parametric model combining physical-behavioral dynamics, explicitly parameterized by fault location/impedance. Confidence interval analysis of estimated parameters determines fault presence, validated via EMTP-RV simulations on a 4-terminal grid. The approach achieves fault localization within 0.5 ms, enabling ultrafast isolation to enhance HVDC grid reliability.

[18] proposed a deep learning-based fault classification method for small-current grounded distribution systems. In this approach, a time-frequency energy matrix was constructed by applying Hilbert-Huang transform (HHT) bandpass filtering to the acquired fault signals. The resulting time-frequency energy matrix serves as the pixel matrix for a digital image, which is then analyzed using an image similarity recognition method based on CNN for fault classification. This method effectively extracts the features of the fault signals, while accurately classifying 10 types of short-circuit faults.

SHAIKH M S et al. [19] developed a Hybrid Grey Wolf-Particle Swarm Optimization (HGWPSO) algorithm to enhance the performance for solving complex engineering optimization problems. By synergistically combining the global exploration of GWO with the fast convergence of PSO, the algorithm was proven effective across various benchmark tests and real-world engineering cases.

SHAIKH M S et al. [20] developed a hybrid MFO-PSO algorithm for estimating transmission line parameters. This method enhances global search capability by integrating the mechanisms of both algorithms. Validation results demonstrated its superior performance over standard PSO and MFO in terms of both solution quality and convergence speed.

LATIF S A et al. [21] proposed a Q-learning-based intelligent duty cycle MAC protocol (RiD-MAC) to address energy efficiency challenges in WSN-enabled IoT applications. Through extensive simulations conducted on the OMNeT++ platform using the Castalia simulator, the protocol was shown to reduce receiver energy consumption by up to 21% and receiver energy consumption per bit by up to 26% compared to state-of-the-art protocols, demonstrating significant energy efficiency improvements.

### Contribution of this study

Despite their widespread application, conventional Convolutional Neural Networks (CNNs) exhibit inherent limitations when applied to the complex task of HVDC fault diagnosis. As network depth increases to capture more intricate features, they often suffer from performance saturation and gradient degradation, ultimately constraining their diagnostic accuracy and robustness. Furthermore, the process of manual feature engineering or complex data preprocessing often required by these models can hinder training efficiency and practical deployment.

To overcome these challenges, this study proposes an improved one-dimensional Residual Neural Network (1D-ResNet). The core of our approach lies in two key innovations: firstly, the adoption of a dual-branch residual structure that facilitates unimpeded gradient flow through identity mappings, enabling the construction of a deeper and more powerful network. Secondly, we integrate a lightweight attention mechanism into the residual blocks, empowering the model to adaptively focus on the most discriminative temporal features within the fault signals, thereby enhancing feature representation and stabilizing the learning process.

### HVDC transmission system structure

HVDC transmission systems can enable the stable interconnection of two independent HVAC systems without requiring synchronization, thereby resolving issues corresponding to the varying connection phases between AC systems. An HVDC system primarily comprises a converter station and a DC line, with the converter station containing components such as a converter transformer, converter, AC filter, and leveling reactor. Among these, the converter is the most critical component; it typically comprises thyristors and functions to perform rectification and inversion, thereby facilitating conversion between AC and DC.

**Fig 1:**
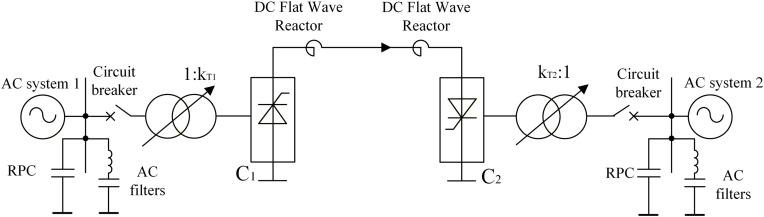
Basic electrical wiring structure of unipolar earth return dc transmission system.

The HVDC transmission system can be divided into unipolar, bipolar, and homopolar systems based on the polarity configuration of the DC conductor. Fig 1 depicts the basic electrical wiring configuration of a unipolar earth-return DC transmission system. DC transmission involves the transfer of electrical energy through a converter that first rectifies the electrical energy from AC to DC, and then inverts it from DC back into AC to flow into an AC system. When electrical energy is transmitted from AC system 1 to AC system 2 via a DC transmission line, Converter $C_{1}$ operates in a rectified state, whereas Converter $C_{2}$ operates in an inverted state.

### Residual neural network

**Fig 2:**
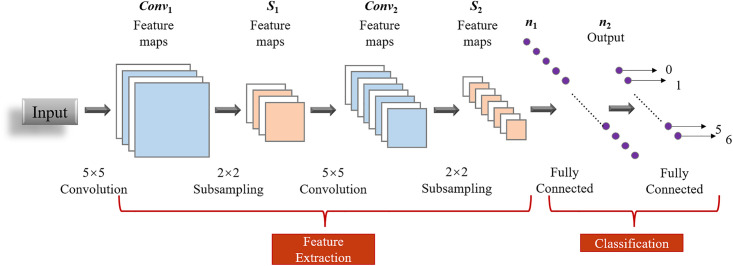
Standard convolutional neural network structure diagram.

The CNN was first proposed by LeCun and is characterized by two key features: weight sharing and pooling [22]. It is a type of feed-forward neural network that incorporates convolutional operations and a deep structure, making it an effective representative algorithm for deep learning. A typical CNN comprises five main components: the input layer, convolutional layer, pooling layer, fully connected layer, and output layer. The input layer receives the raw data, followed by multiple convolutional and pooling layers that extract the required features and reduce dimensionality. Lastly, the fully connected layer further refines the features before they are passed to the output layer for classification. Fig 2 depicts the standard structure of a convolutional neural network. One-dimensional and two-dimensional convolution exhibit a similar structural framework, with the key distinction being that the one-dimensional convolution kernel slides in a single direction to extract features from the one-dimensional data, thereby enabling classification.

**Fig 3:**
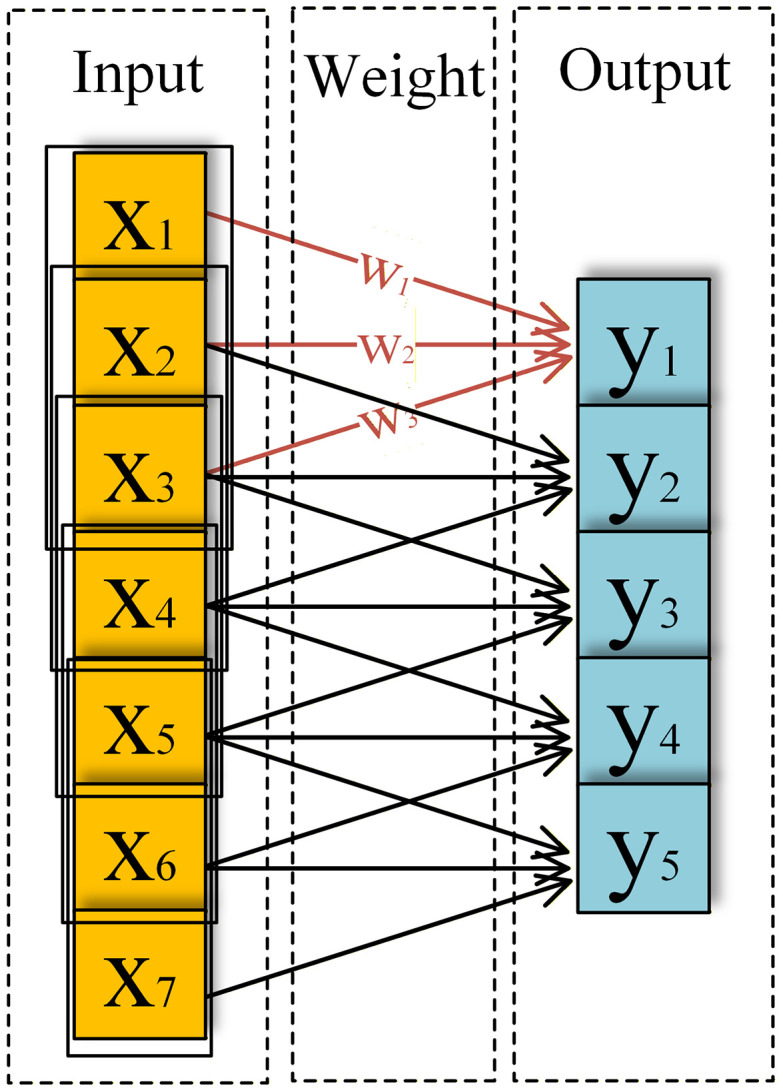
One-dimensional convolutional operation process diagram.

In the convolutional layer, a convolutional kernel performs convolution on the input signals received from the input layer, presenting feature maps that highlight different data characteristics [23]. Weight sharing, a key attribute of the convolutional layer, enables parameter reuse, which significantly reduces the number of parameters, which enhances the generalization ability of the model and mitigates the risk of overfitting. The specific operational process of the convolutional layer is expressed as follows.


$$y^{l}=\sum_{i=1}^{c^{l-1}}w_{i,c}^{l}*x_{i}^{l-1}+b_{i}^{l}$$ (1)

where, $y^{l}$ denotes the output of layer l; $c^{l-1}$ denotes the cth channel of layer l−1; $w_{i,c}^{l}$ denotes the convolutional kernel parameter weight matrix of layer l, where i denotes the ith channel in layer l and c denotes the cth channel in layer l−1; * denotes the convolutional operation; $x_{i}^{l-1}$ denotes the output, x, of the ith channel in the layer l−1; and $b_{i}^{l}$ denotes the bias term. Fig 3 depicts the specific operation procedure.

**Fig 4:**
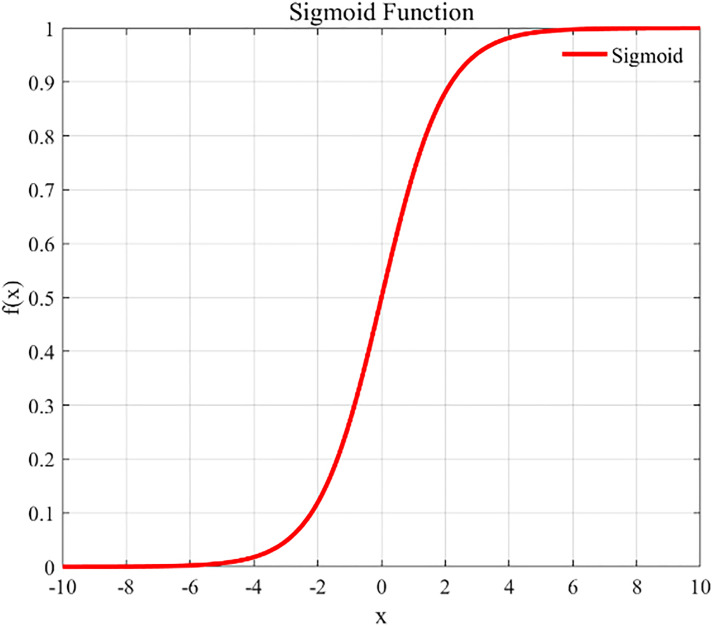
Sigmoid.

**Fig 5:**
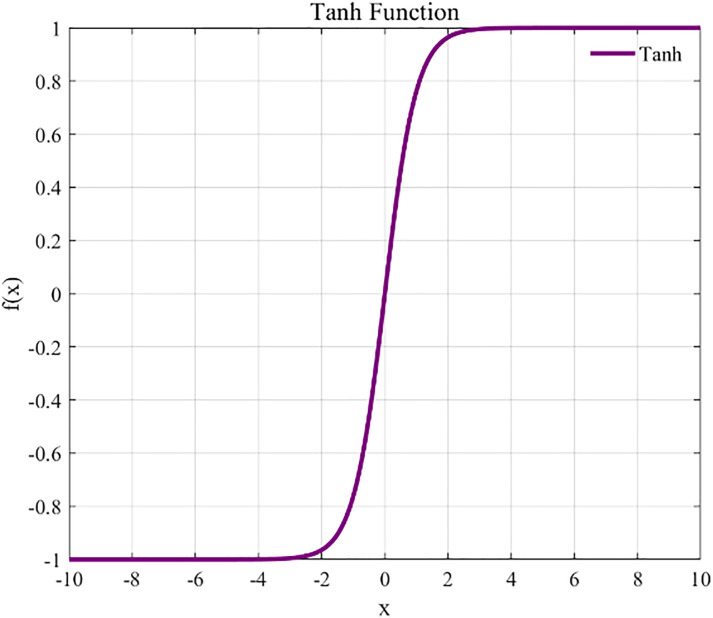
Tanh.

**Fig 6:**
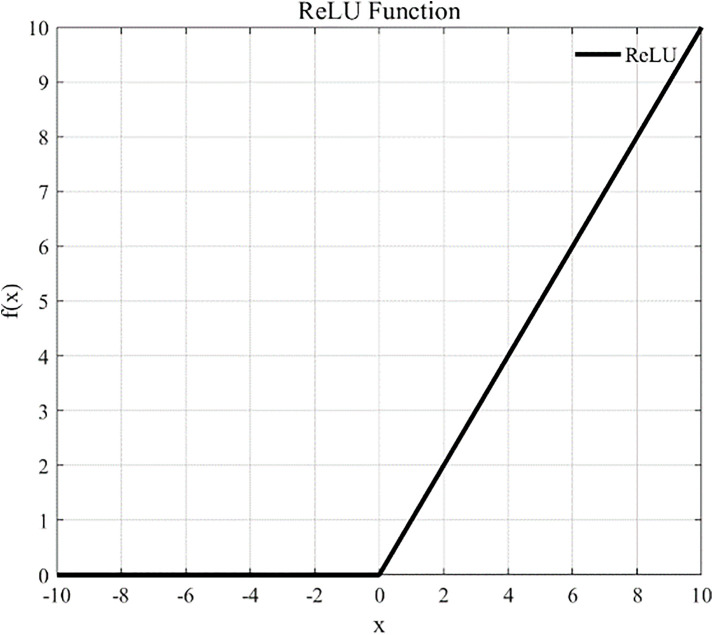
ReLU.

Following the convolution operation, we employed an activation function to subject the output of the convolutional layer to a nonlinear transformation, which maps the features that were nonlinearly inseparable into a space where the linear separability is enhanced. The commonly used activation functions include the Sigmoid function, hyperbolic tangent (Tanh)function, and linear rectifier (ReLu) function. The specific expressions for these three activation functions are presented below:


$$Sigmoid(y^{l(i,j)})=\frac{\text{1}}{1+e^{-y^{l(i,j)}}}$$ (2)

$$Tanh\mathrm{\backslash nolimits}(y^{l(i,j)})=\frac{e^{y^{l(i,j)}}-e^{-y^{l(i,j)}}}{e^{y^{l(i,j)}}+e^{-y^{l(i,j)}}}$$ (3)

$$Re\mathrm{\backslash nolimits}LU(y^{l(i,j)})=\max\{0,y^{l(i,j)}\}$$ (4)

where $y^{l(i,j)}$ denotes the jth eigenvalue of the ith eigenmap in the lth convolutional layer. Figs 4–6 depict the graphs of these three activation functions, respectively. Among them, the ReLu function accelerates the convergence of the model training process, and its derivative equals 1 for inputs greater than 0, which helps in effectively overcoming the vanishing gradient problem. Therefore, we selected the ReLu function as the activation function for the neural network.

**Fig 7:**
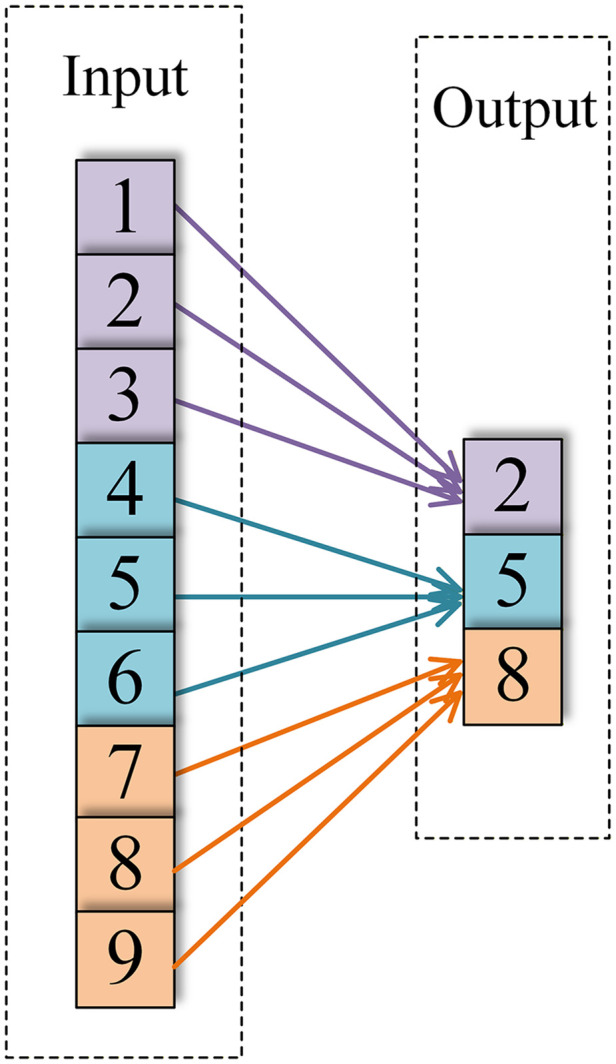
Average pooling.

**Fig 8:**
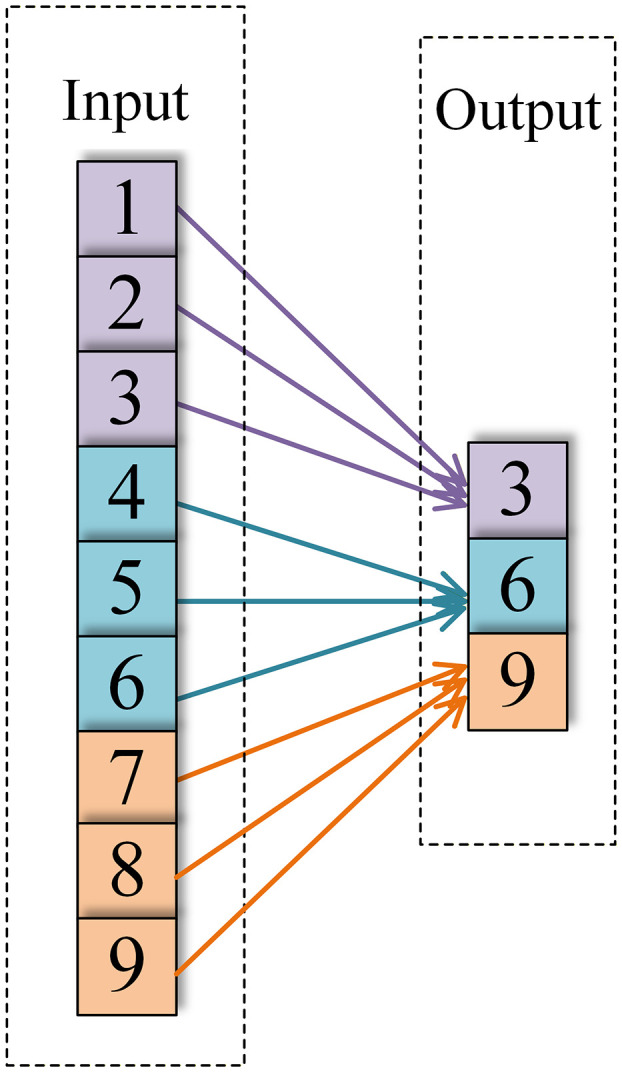
Maximum pooling.

In CNNs, the pooling layer compresses the feature dimensions to reduce the computational load; this process is known as down-sampling. The pooling layer is typically positioned after the activation layer in one-dimensional CNNs, which employ two types of pooling operations: average pooling and max pooling. They are expressed as follows:


$$y^{l(i,j)}=\frac{\text{1}}{W}\sum_{t=(j-1)W+1}^{jW}a^{l(i,t)}$$ (5)

$$y^{l(i,j)}=\underset{(j-1)W+1\leq jW}{\max}\{a^{l(i,t)}\}$$ (6)

where $y^{l(i,j)}$ denotes the output value of the jth neuron in the ith channel of the lth layer, $a^{l(i,t)}$ denotes the input value of the tth neuron in the ith channel of the lth layer, and W denotes the width of the pooling kernel. Fig 7 and Fig 8 depict the specific operation procedures of the one-dimensional average pooling operation and one-dimensional maximum pooling operation, respectively.

The fully connected layer in a CNN integrates the features extracted from the convolutional and pooling layers into global features for classification and recognition. The output from the final pooling or convolutional layer is flattened into a one-dimensional feature vector. Subsequently, the output is then fed into the fully connected layer for further feature extraction and refinement, thereby significantly enhancing the nonlinear representation of the features. The specific operational formula for the forward propagation process in the fully connected layer is given as follows:


$$y^{l}=\sum_{i=1}^{n}w_{ij}^{l-1}x^{l-1(i)}+b_{i}^{l-1}$$ (7)

where $y^{l}$ denotes the output vector, y denotes the fully connected layer l, $x^{l-1(i)}$ denotes the output value of layer l−1, $w_{ij}^{l-1}$ denotes the weights between the ith neuron of layer l−1 and the jth neuron of layer l, and $b_{i}^{l-1}$ denotes the bias of the jth neuron of layer l−1.

The performance of CNNs may deteriorate as the network depth increases. This phenomenon can be attributed to the fact that stacking many layers can exacerbate issues such as vanishing and exploding gradients, thereby degrading the effectiveness of the network training. These challenges can be effectively mitigated by using the residual network (ResNet) proposed by He et al. in 2015 [24].

**Fig 9:**
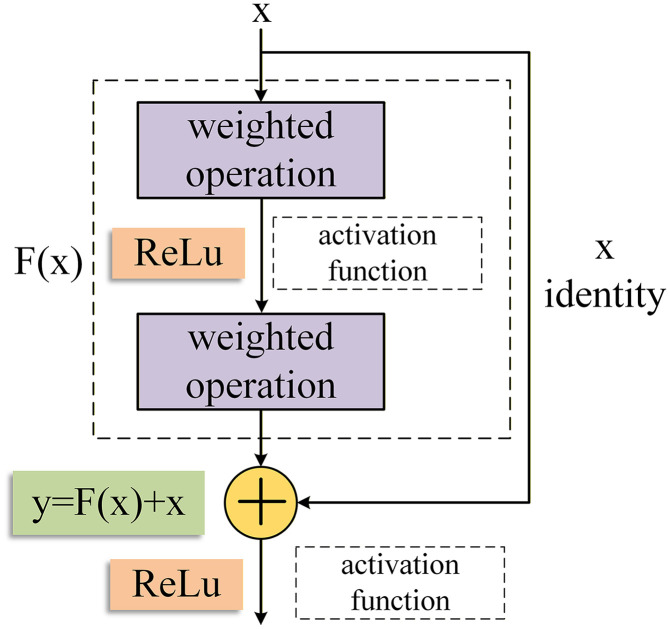
Schematic structure of the residual module.

Residual neural networks can enhance the performance of conventional CNNs by incorporating residual connection modules. ResNet can directly forward the output of the current layer to the next layer through constant mapping, thereby effectively creating a shortcut that bypasses the transformation of the current layer. During BP, this shortcut also enables the gradient from the subsequent layer to be passed directly to the preceding layer. Fig 9 depicts a schematic of the structure of the residual block, where x denotes the input of the residual block, y denotes its output, and F(x) represents the residual mapping to be learned. The relationship, y = F(x)+x, holds due to the constant mapping, implying that the module only needs to learn the residual function, F(x)=y − x.

During the training process, the input features exhibit varying sensitivities to faults, and different fault types rely on distinct key features. We introduced the channel attention mechanism module to optimize the network performance by enabling the network to automatically learn the importance weights of different feature channels, enhancing the representation of key feature channels, and suppressing irrelevant or noisy channels [25].

Step 1: Squeeze

The timing features of each channel are compressed into a scalar statistic through global average pooling. This scalar statistic characterizes the global importance of that channel, and is expressed as follows:


$$z_{c}=\frac{\text{1}}{T}\sum_{t=1}^{T}x_{c}^{\left(t\right)}$$ (8)

where $z_{c}$ denotes the global average of the cth channel, which represents the overall activity of the channel in the time dimension and T =2000 denotes the total number of time steps corresponding to 1.4 seconds of sampling points of one.

Step 2: Excitation

This process employs two fully connected layers, wherein the dynamic weights are generated by learning the nonlinear relationships between the channels through these layers. It is expressed as follows:


$$s=\sigma (W_{2}\cdot \delta (W_{1}\cdot z))$$ (9)

where z denotes the global feature vector output obtained from the Squeeze stage; $W_{1}$ denotes the descending matrix; $W_{2}$ denotes the ascending matrix; σ denotes the Sigmoid function, which normalizes the weights to [0,1]; and δ denotes the ReLU activation function, which enhances the nonlinearity.

Step 3: Reweight

**Fig 10:**
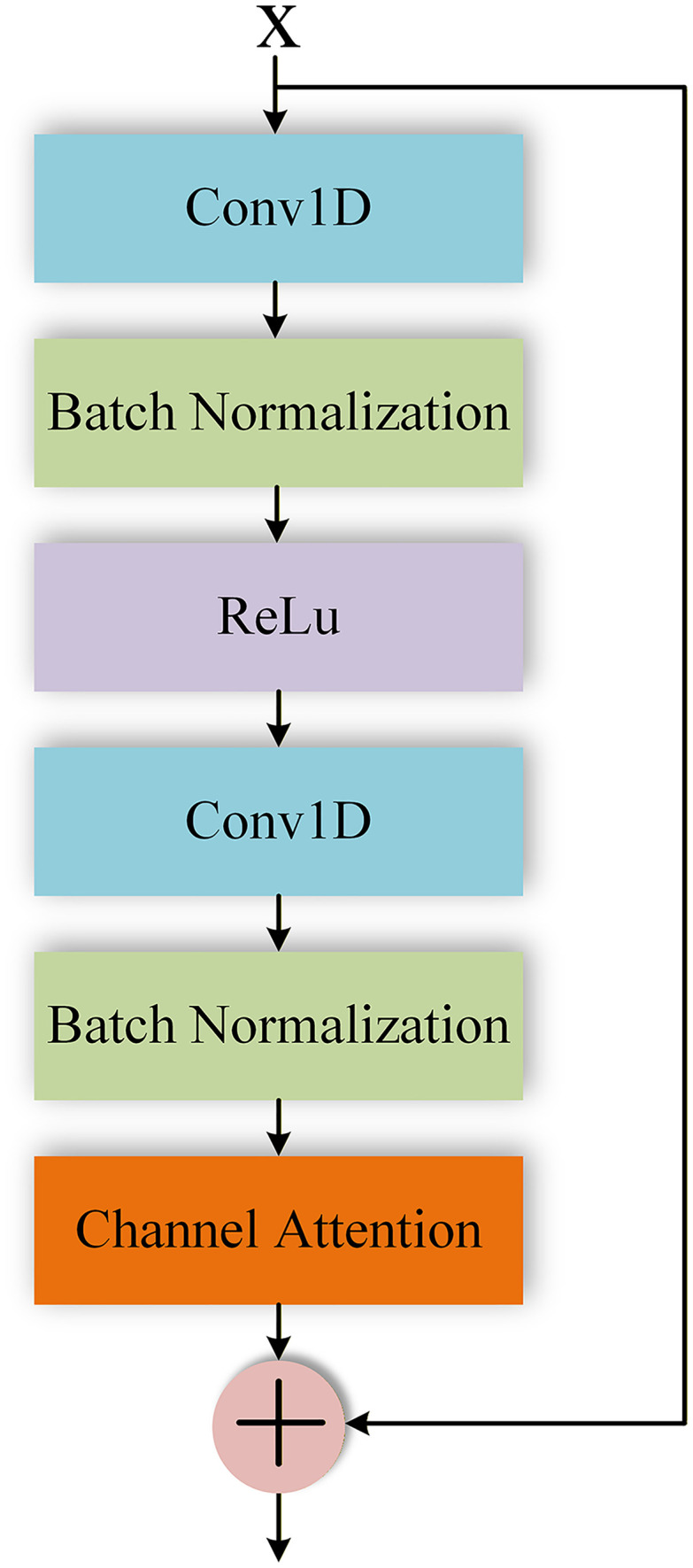
Improved residuals module diagram.

The weights are assigned to amplify the signal amplitude of the key channels by converging their weights to 1, which ensures that they dominate the subsequent network and enhance the feature representation. Conversely, the influence of non-key channels is suppressed by converging their weights to 0, which reduces their impact and improves the robustness of the model against interference. The mathematical expression is given as follows:


$$X_{out}=s⊙X_{in}$$ (10)

where $X_{out}$ denotes the reweighted feature tensor and ⊙ denotes the channel-by-channel multiplication. Fig 10 depicts the schematic of the improved residual module.

### Fault diagnosis model based on residual neural network

**Fig 11:**
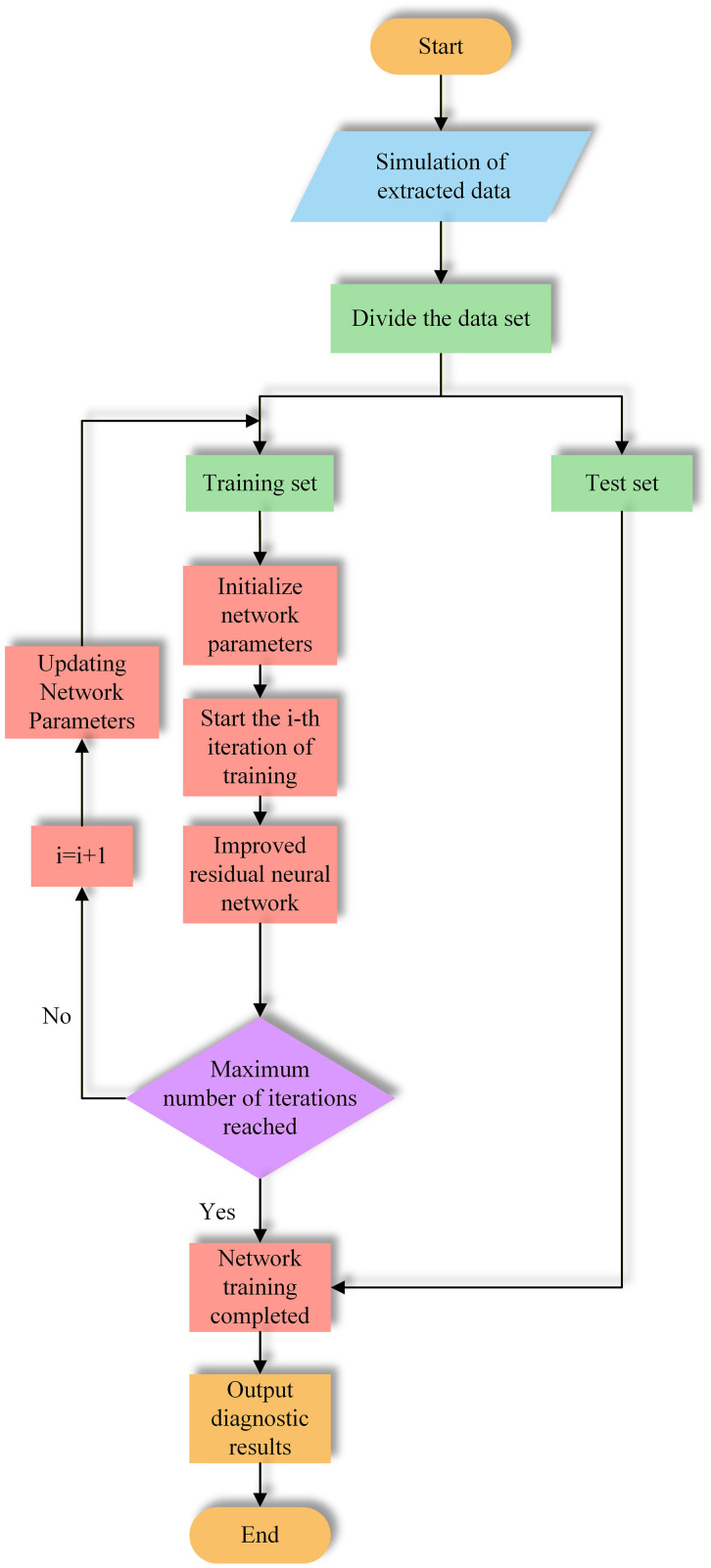
Diagram of the diagnostic process of residual neural network.

Conventional CNNs may face issues such as gradient vanishing with the increase in the number of layers. Therefore, we adopted an improved residual neural network to replace the conventional CNNs for training. Fig 11 depicts the fault diagnosis process based on the improved residual neural network, and outlines the main steps involved.

(1) Build data set: The simulation model of the high-voltage DC transmission system is first established in MATLAB Simulink. The fault conditions are then configured and executed individually. The acquired data were extracted using the To Workspace function to form a complete dataset. Lastly, the dataset is partitioned into a training set and a testing set in a ratio of 7:3.
(2) Model training: The residual neural network is constructed in MATLAB and then improved. The training set data are fed into the enhanced residual neural network model for training. The improved residual block effectively mitigates the problems of gradient vanishing and explosion. The network parameters are updated using the BP algorithm, and the training process continues until the maximum number of iterations is reached.
(3) Model testing: The test set data are fed into the trained network model for evaluation, which helps in evaluating the model training performance.

### Example analysis

**Fig 12:**
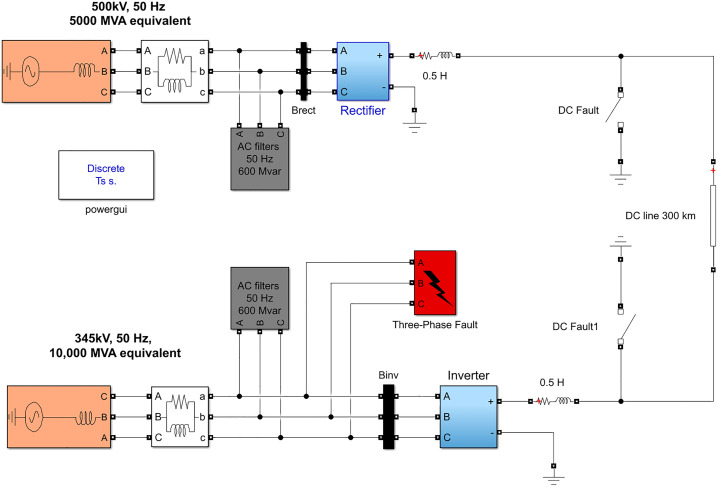
System diagram of the simulation model of high-voltage direct current transmission system.

To verify the effectiveness of the model in fault diagnosis of HVDC transmission systems, we constructed a HVDC transmission system model using MATLAB/Simulink, as shown in Fig 12.

Fig 12 depicts the simulation model of the HVDC transmission system, where the power is delivered from a 500 kV, 5000 MVA, and 50 Hz power system (EM) to a 345 kV, 1000 MVA, and 50 Hz power system (EN) via a 1000 MW (500 kV, 2 kA) DC transmission line. In this system, the AC filtering section is directly connected to the AC bus and includes branch circuits for the 11th, 13th, and higher-order harmonics, providing a capacity of up to 600 MVAR. Both the rectifier and inverter sections are constructed using two generalized 6-pulse bridges. Table 1 lists the parameters of the DC transmission line.

**Table 1:** DC transmission line parameters.

Line resistance	Line inductor	Line capacitor	Line length
0.015Ω/km	0.792mH/km	14.4nF/km	300km

We consider seven typical states during the simulation process: normal operation, DC line-to-ground fault at the rectifier end, DC line-to-ground fault at the inverter end, single-phase grounding, two-phase grounding, three-phase grounding, and two-phase inter-phase short circuit. The seven fault characteristics represent the DC voltage of the rectifier terminal, DC current of the rectifier terminal, DC voltage of the inverter terminal, DC current of the inverter terminal, and the AC currents of phase A, B, and C, respectively. When simulating single-phase and two-phase ground faults, selecting any one of the three AC phases (A, B, or C) or any two phases (A and B, B and C, or C and A) produces the same effect on the DC voltage and current, whereas the AC waveform effectively identifies the faulty phase. Therefore, phase A is selected as the faulty phase for the single-phase ground fault simulations, phases A and B for the two-phase ground fault simulations, and phases A and B for the two-phase inter-phase short circuit fault simulations. Table 2 depicts the fault data descriptions.

**Table 2:** HVDC system fault data description table.

Fault state	Training/Testing samples	Label
Normal state	1400/600	0
DC line ground at rectifier	1400/600	1
DC line ground at inverter	1400/600	2
Single-phase grounding	1400/600	3
Two-phase grounding	1400/600	4
Three-phase grounding	1400/600	5
Short circuit between two phases	1400/600	6

The entire simulation time is set to 1.4 s, and all the faults are set to 0.7 s-0.8 s. The time step of the simulation is set to 50 μs. Therefore, 2000 time points of the data can be obtained in each state in the entire simulation process, thereby obtaining a total of 14000 sets of data.

When applied to specific classification tasks, the performance of the residual neural network model can be demonstrated more effectively by optimizing its parameters. Consequently, Table 3 lists the training parameters of the residual network.

**Table 3:** Residual neural network model parameters.

Network layer	Parameters	
Input layer	Input data:7 × 1 × 1	
Initial Convolutional layer	Convolution kernel:3 × 3, Stride:1	
Activation layer	ReLu	
Maximum pooling layer	Pooling window:1 × 1, Stride:2	
Residual module×3	Convolutional layer	Convolution kernel:3 × 3
		Quantity: 64
		Stride: 1
		Filling: same
	Activation layer	ReLu
	Addition layer	Adding two inputs
Full connectivity layer	Number of output nodes: 7	
SoftMax layer	Number of nodes: 7	

In terms of the other parameters, we selected the Adam optimizer since it adaptively adjusts the learning rate of each parameter by computing the first and second-order moment estimates of the gradient, thereby effectively mitigating the issues associated with a fixed global learning rate. The initial learning rate is set to1e-3, batch size is set to 64, maximum number of iterations is set to 150, L2 regularization parameter is set to 1e-4, and the cross-entropy loss function is employed.

To verify the diagnostic effectiveness of the residual neural network-based fault diagnosis method presented in this paper, we first compare networks with different numbers of iterations and different optimizers. We then evaluate the diagnostic accuracy, training loss, and classification accuracy corresponding to a conventional CNN.

The number of iterations is a critical parameter in network training. We determined the optimal number of iterations by varying the iteration count and evaluating training performance. Table 4 summarizes the training outcomes of the network model for different iteration numbers. It can be observed that the model accuracy improves with the increase in the number of iterations increases; however, the training time also increases correspondingly. An accuracy of 99.15%is achieved when the number of iterations reaches 150. Although the accuracy is similar for 150, 200, and 250 iterations, the training time increases significantly. In particular, when the number of iterations increases from 150 to 200, the training time increases by 201.9 s, and at 250 iterations, only a marginal improvement in the accuracy is achieved while the training time more than doubles when compared with that for 150 iterations. Therefore, we ultimately selected 150 iterations as the maximum number for the network training.

**Table 4:** Impact of different number of iterations on modeling.

Number of iterations	Accuracy (%)	Training time(s)
30	82.64	105.13
90	95.03	221.35
150	99.15	423.25
200	99.26	751.67
250	99.51	1023.17

Optimizer algorithms in neural networks adjust the network parameters to minimize the loss function and improve the model accuracy. During training, the optimizer updates the parameters to ensure that the model achieves better optimization. Various optimizer algorithms can be employed, each with its own advantages and drawbacks in adjusting the model parameters. Therefore, we selected the optimal optimizer by comparing the effects of several common algorithms on the model training. Table 5 summarizes the training outcomes of the model under different optimizer algorithms. It can be observed that while the SGD optimizer achieves an accuracy of only 89.47%, the Adam optimizer achieves an accuracy of 99.15%. Furthermore, the time required for the model to converge is significantly lower when using the Adam optimizer, only 423.25 s, when compared with the other optimizers that require considerably longer convergence time. The Adam optimizer achieves the best performance based on the combined accuracy and training time; hence, we adopted the Adam algorithm to optimize the network parameter updates.

**Table 5:** Effect of different optimizers on model training.

Optimizer	Accuracy (%)	Training time (s)
Adam	99.15	423.25
SGD	89.47	516.13
Momentum	92.16	463.16
RMSProp	96.88	468.65
Adagrad	93.24	430.15

**Fig 13:**
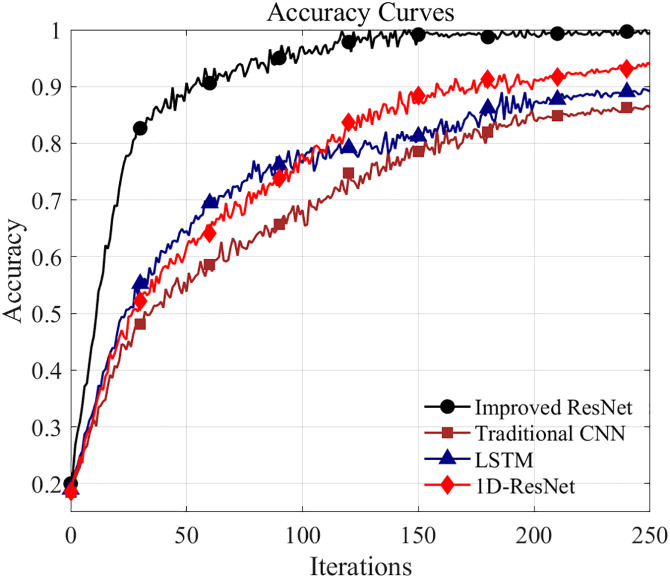
Comparison of accuracy curve.

**Fig 14:**
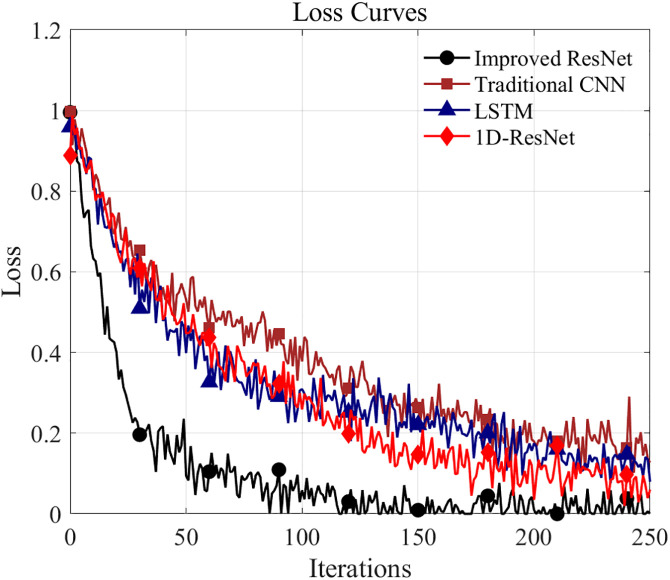
Comparison of loss ratio curves.

To further validate the superiority of the proposed improved ResNet model, a comprehensive comparison was conducted with three baseline models: traditional CNN, LSTM, and 1D-ResNet. Fig 13 and Fig 14 depict the comparison curves for diagnostic accuracy and training loss, respectively. It can be observed that the proposed improved ResNet achieves an accuracy of over 80% within 30 iterations, significantly outperforming the traditional CNN (approximately 45%), LSTM (approximately 55%), and 1D-ResNet (approximately 52%). At the end of training, the proposed method achieves a diagnostic accuracy of 99.15%, demonstrating substantial improvements over the conventional CNN (86.26%), LSTM (89.14%), and 1D-ResNet (93.83%). Furthermore, in terms of training loss, the loss curve of the proposed method declines more rapidly than those of all three baseline models, with a final loss of only 0.03 compared to 0.18 for traditional CNN, 0.13 for LSTM, and 0.08 for 1D-ResNet. These results demonstrate that the proposed improved ResNet method effectively enhances diagnostic accuracy while reducing training loss when compared with conventional CNN, LSTM, and 1D-ResNet approaches.

**Fig 15:**
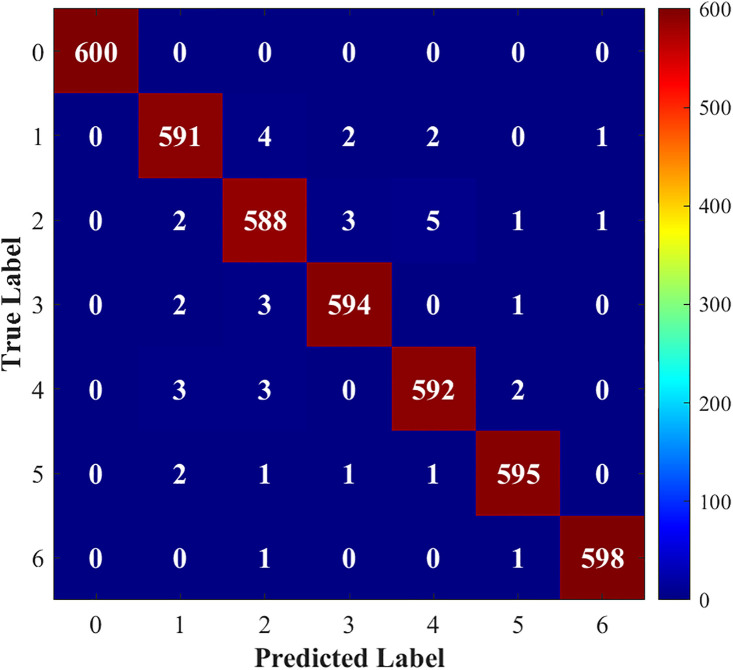
Improved ResNet.

**Fig 16:**
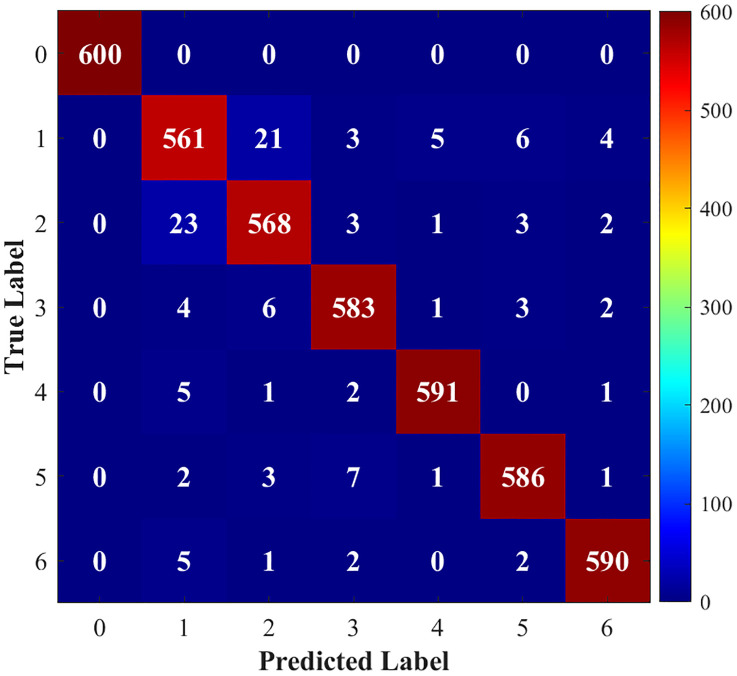
Traditional CNN.

**Fig 17:**
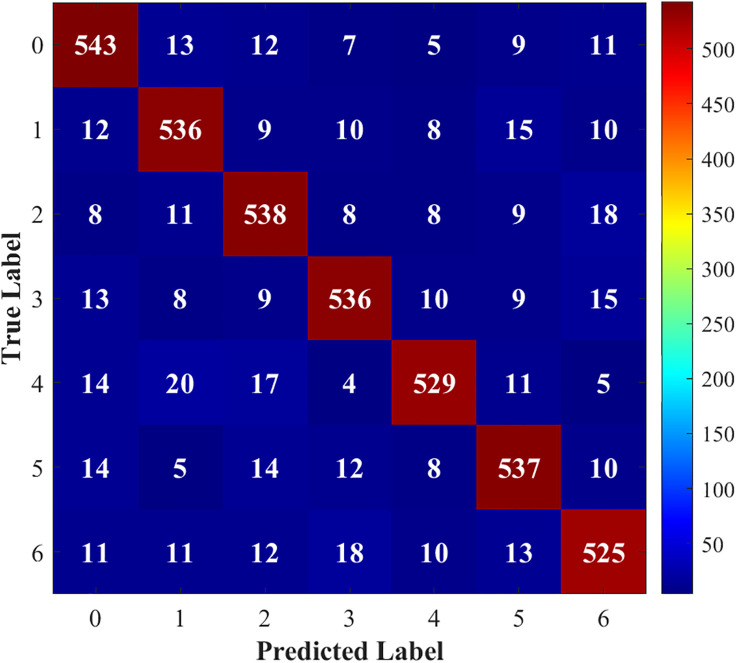
LSTM.

**Fig 18:**
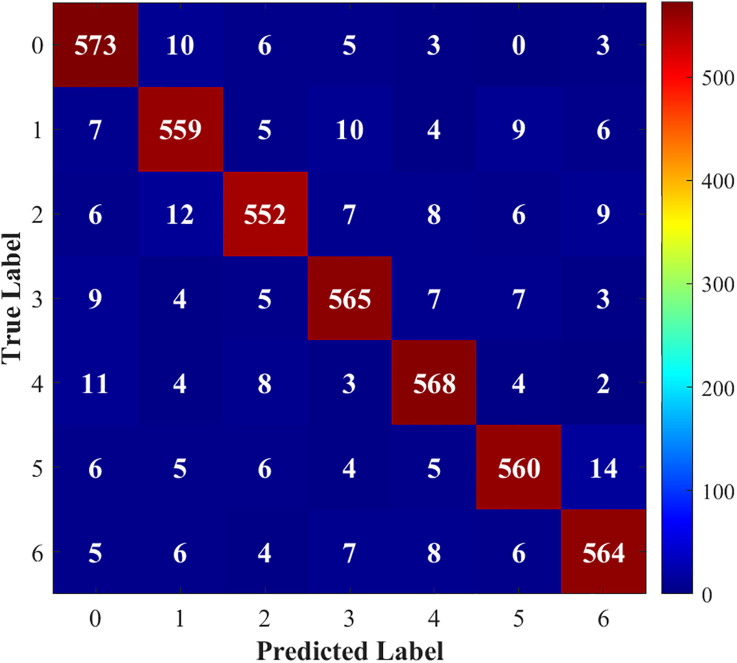
1D-ResNet.

Figs 15–18 show the confusion matrix drawn after fault diagnosis through four methods. Notably, the proposed method achieves the highest classification accuracy for faults on the DC line at the rectifier end, whereas the accuracies of the two methods for two-phase ground contact faults are similar. Overall, the comparison demonstrates that the residual network diagnosis method outperforms the conventional CNN in terms of the classification accuracy across various faults.

To assess the statistical reliability and stability of the reported diagnostic accuracy, we performed a 95% confidence interval (CI) analysis based on repeated independent training runs. Specifically, the proposed model was trained and evaluated 10 times using different random seeds, which affected weight initialization and minibatch ordering. Let $A_{1}$, $A_{2}$, …, $A_{10}$ denote the accuracies obtained from these experiments. The sample mean $\bar{A}$ and sample standard deviation s were computed as:


$$\bar{A}=\frac{\text{1}}{N}\sum_{i=1}^{N}A_{i}$$ (11)

$$s=\sqrt{\frac{\text{1}}{N-1}\sum_{i=1}^{N}{\left(A_{i}-\bar{A}\right)}^{2}}$$ (12)

where N = 10. A Student’s t-based 95% confidence interval for the mean accuracy was obtained using:


$$CI_{95\%}=\bar{A}\pm t_{N-1,0.975}\cdot \frac{s}{\sqrt{N}}$$ (13)

with $t_{9,0.975}$ =2.262. In addition, a non-parametric bootstrap CI was computed using 5,000 resampling iterations from the accuracy samples $A_{i}$.The 2.5th and 97.5th percentiles of the bootstrap distribution were used as CI boundaries. This dual analysis provides a robust estimate of uncertainty in the reported diagnostic accuracy.

**Fig 19:**
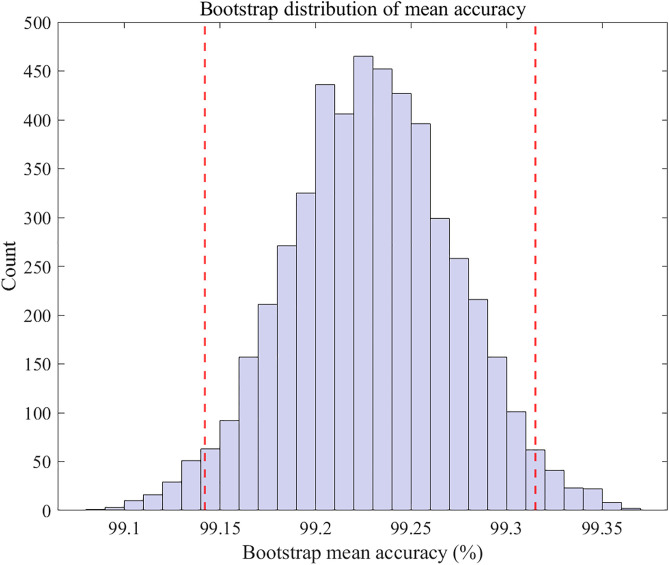
Bootstrap distribution of mean accuracy.

To quantify the reliability of the proposed method, we evaluated the accuracy over 10 independent runs. The sample mean accuracy is $\bar{A}$ =99.15%, and the sample standard deviation is s =0.32%. Using the t-based method, the CI for the mean accuracy is: [98.92%,99.38%]. A bootstrap-based non-parametric CI yielded a consistent interval of: [99.15%,99.32%]. The narrow width of both intervals demonstrates that the proposed attention-enhanced ResNet exhibits high stability and low performance variance across repeated training runs. The bootstrap distribution of the mean accuracy is shown in Fig 19.

Fig 19 illustrates the bootstrap distribution of the mean diagnostic accuracy obtained from 5,000 resampling iterations. Each bar represents the frequency of bootstrap sample means computed by repeatedly sampling from the accuracies obtained across 10 independent runs. The distribution is approximately normal, indicating stable performance across repeated trials. The two red dashed lines denote the 95% confidence interval boundaries (2.5th and 97.5th percentiles), which in this case fall within approximately 99.15% and 99.32%. The narrow interval demonstrates that the proposed model exhibits very low variance and high reliability in diagnostic performance.

The practical deployment of a fault diagnosis model in HVDC protection systems necessitates not only high accuracy but also stringent computational efficiency to meet real-time operational requirements. To evaluate this critical aspect, we conducted a comprehensive computational latency analysis of our proposed improved 1D-ResNet model and compared it against the baseline models.

The benchmarking was performed on two platforms representative of potential deployment environments: (1) a high-performance CPU (Intel Xeon E5-2680 @ 2.5GHz) simulating a central monitoring server, and (2) an embedded GPU (NVIDIA Jetson Xavier NX) representing a potential edge-computing device within a protection relay. The models were tested using a batch size of 1 to simulate the real-world scenario of processing a single data window from a continuous stream. For each model, the average inference time per sample was calculated over 1,000 independent runs after a 100-run warm-up to eliminate cold-start effects.

The results are summarized in Table 6. Our proposed improved 1D-ResNet model achieved an average inference time of 0.82 ms on the embedded GPU and 4.15 ms on the server CPU. This performance is critical, as typical HVDC line protection schemes require decision-making within 1–5 milliseconds. Although our model has a higher parameter count (5.2M) than the conventional CNN (2.1M), its architectural efficiency allows it to outperform the standard 1D-ResNet (4.5M) in terms of speed, thanks to the optimized use of attention mechanisms and bottleneck layers. The LSTM model, as expected, exhibited significantly higher latency due to its sequential processing nature, rendering it less suitable for high-speed protection applications.

**Table 6:** Computational performance benchmarking.

Model	Parameters (M)	Inference time – CPU (ms)	Inference time – GPU (ms)	Throughput – GPU (samples/s)
Conventional CNN	2.1	2.91 ± 0.23	0.51 ± 0.05	1960
Standard 1D-ResNet	4.5	5.82 ± 0.41	1.12 ± 0.08	893
Proposed Improved 1D-ResNet	5.2	4.15 ± 0.31	0.82 ± 0.07	1219
LSTM	3.8	15.67 ± 1.25	3.45 ± 0.29	290

**Fig 20:**
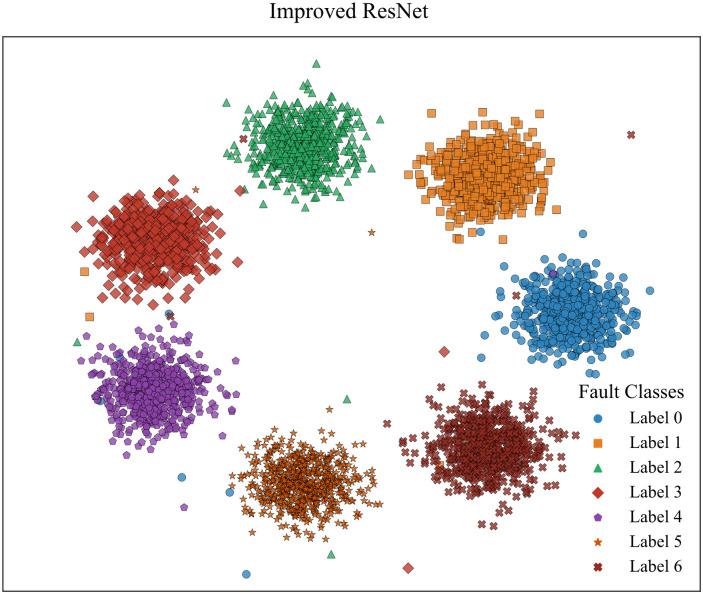
Feature visualization of improved residual network.

**Fig 21:**
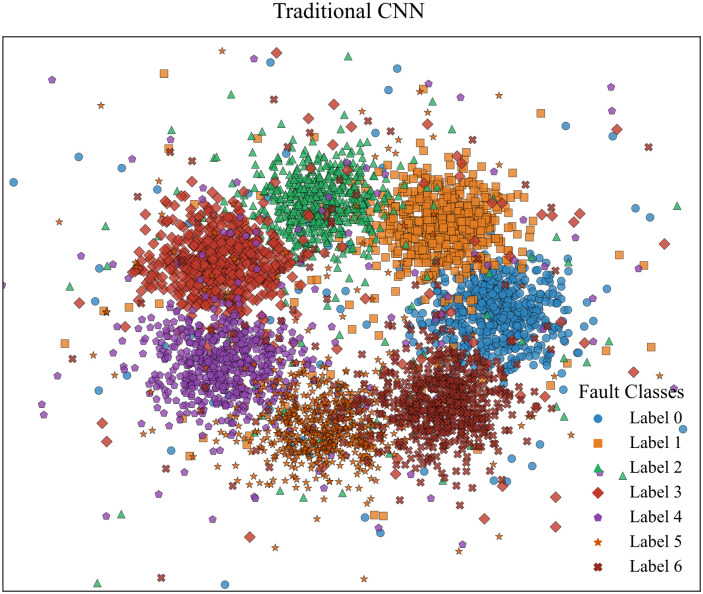
Feature visualization of traditional CNN.

To visualize the feature extraction capability of the improved ResNet, we employed t-SNE to project the feature outputs from the final fully connected layer of both the improved ResNet model and the conventional CNN model. Fig 20 and Fig 21 depict the resulting feature visualization plots. It can be observed that after feature aggregation, the improved ResNet distinctly separates the fault features of different fault types with minimal intra-class distances and only a few misclustered samples. Conversely, the feature visualization of conventional CNN demonstrates several fault features positioned near the decision boundaries that are not accurately classified. This comparison clearly demonstrates that the proposed method exhibits superior feature extraction capability.

## Conclusion

In this study, we employed a residual neural network for the fault diagnosis of HVDC transmission systems. The network was trained and tested using the data obtained from a simulation model, and its performance is compared with that of conventional CNN algorithms. The following conclusions are drawn:

(1) The proposed fault diagnosis method based on the improved one-dimensional residual neural network can be trained directly by using the simulation data as the input. This approach simplifies data preprocessing, improves the training efficiency, and significantly reduces the training time.
(2) When compared with conventional CNNs, the proposed ResNet incorporates a dual-branch structure within each residual module, followed by feature fusion and nonlinear activation after branch computation. Furthermore, an attention mechanism is integrated into the residual module, which enhances the gradient propagation and improves the parameter optimization, thereby effectively reducing the risk of error bias during the model training.
(3) Experimental evaluation confirms the efficacy of the proposed model, which attains an average diagnostic accuracy of 99.15%, significantly exceeding that of LSTM (89.14%), conventional CNN (86.26%), and the standard 1D-ResNet (93.83%). This performance hierarchy underscores the contribution of our architectural improvements. Moreover, the model converges to a substantially lower training loss, demonstrating superior robustness and more stable convergence behavior.

In summary, the fault diagnosis method based on the proposed one-dimensional residual neural network demonstrates promising application prospects in HVDC transmission systems, and can provide effective technical support for intelligent fault identification under complex operating conditions.

### Nomenclature

**Table:** 

Abbreviation	Description
**AI**	Artificial Intelligence
**ANN**	Artificial Neural Network
**1D-CNN**	One-Dimensional Convolutional Neural Network
**1D-ResNet**	One-Dimensional Residual Neural Network
**AC**	Alternating Current
**Adam**	Adaptive Moment Estimation (an optimizer)
**AI**	Artificial Intelligence
**ANN**	Artificial Neural Network
**BPNN**	Back Propagation Neural Network
**CNN**	Convolutional Neural Network
**DC**	Direct Current
**GRU**	Gated Recurrent Unit
**Grad-CAM**	Gradient-weighted Class Activation Mapping
**HHT**	Hilbert-Huang Transform
**HVDC**	High-Voltage Direct Current
**LSTM**	Long Short-Term Memory
**PSO**	Particle Swarm Optimization
**ReLU**	Rectified Linear Unit (an activation function)
**RF**	Random Forest

## References

[1] Xiang W, Yang S, Adam GP, Zhang H, Zuo W, Wen J. DC fault protection algorithms of MMC-HVDC grids: fault analysis, methodologies, experimental validations, and future trends. IEEE Trans Power Electron. 2021;36(10):11245–64. doi: 10.1109/tpel.2021.3071184

[2] Bahrman M, Johnson B. The ABCs of HVDC transmission technologies. IEEE Power and Energy Mag. 2007;5(2):32–44. doi: 10.1109/mpae.2007.329194

[3] Wang T, Song G, Wu L, Wei H, Guang S, Chao L. Novel reclosure scheme of MMC-HVDC system based on characteristic signal injection. J Eng. 2019;2019(16):1153–7. doi: 10.1049/joe.2018.8869

[4] Liu T, Zhang Y, Wang S, Li X, Gooi HB, Ghias AMYM. Fault identification and fault location methods for VSC-HVDC transmission lines based on the traveling waveform difference. Inter J Elect Power Energy Syst. 2023;147:108867. doi: 10.1016/j.ijepes.2022.108867

[5] Yang S, Xiang W, Zhou M, Zuo W, Wen J. A single-end protection scheme for hybrid MMC HVDC grids considering the impacts of the active fault current-limiting control. IEEE Trans Power Delivery. 2021;36(4):2001–13. doi: 10.1109/tpwrd.2020.3017895

[6] Xiao H, Li Y, Liu R, Duan X. Single-end time-domain transient electrical signals based protection principle and its efficient setting calculation method for LCC-HVDC lines. IET Generation Trans Dist. 2017;11(5):1233–42. doi: 10.1049/iet-gtd.2016.1159

[7] Tzelepis D, Dysko A, Fusiek G, Nelson J, Niewczas P, Vozikis D, et al. Single-ended differential protection in MTDC networks using optical sensors. IEEE Trans Power Delivery. 2017;32(3):1605–15. doi: 10.1109/tpwrd.2016.2645231

[8] Yu JJQ, Hou Y, Lam AYS, Li VOK. Intelligent fault detection scheme for microgrids with wavelet-based deep neural networks. IEEE Trans Smart Grid. 2019;10(2):1694–703. doi: 10.1109/tsg.2017.2776310

[9] Choudhary B. An advanced genetic algorithm with improved support vector machine for multi-class classification of real power quality events. Elect Power Syst Res. 2021;191:106879. doi: 10.1016/j.epsr.2020.106879

[10] Sayed Tassawar Hussain K, Song G, Wang T, Hou J, Masood B. Adaptive fault recovery strategy of LCC-MMC based hybrid HVDC. IET Generat Trans Dist. 2021;15(16):2396–409. doi: 10.1049/gtd2.12186

[11] Wang T, Yu Z, Xie F, Hao Z, Monti A, Ponci F. Protection of line faults in HVDC grids through convexity detection in backward traveling wave voltages. IEEE Trans Power Delivery. 2023;38(5):3660–76. doi: 10.1109/tpwrd.2023.3286863

[12] Johnson JM, Yadav A. Complete protection scheme for fault detection, classification and location estimation in HVDC transmission lines using support vector machines. IET Sci Measure Tech. 2017;11(3):279–87. doi: 10.1049/iet-smt.2016.0244

[13] Kou R, Wang Y. Transmission line fault identification based on BP neural network. In: 2019 IEEE innovative smart grid technologies-Asia (ISGT Asia). IEEE; 2019. 991–4.

[14] Moloi K, Yusuff AA. A Wavelet-neural network-based technique for fault diagnostics in power system. In: 2020 7th International conference on soft computing and machine intelligence (ISCMI), 2020. 131–5. doi: 10.1109/iscmi51676.2020.9311601

[15] Wu H, Wang Q, Yu K, Hu X, Ran M. A novel intelligent fault identification method based on random forests for HVDC transmission lines. PLoS One. 2020;15(3):e0230717. doi: 10.1371/journal.pone.0230717 32214364 PMC7098650

[16] Jawad RS, Abid H. HVDC fault detection and classification with artificial neural network based on ACO-DWT method. Energies. 2023;16(3):1064. doi: 10.3390/en16031064

[17] Verrax P, Bertinato A, Kieffer M, Raison B. Fast fault identification in bipolar HVDC grids: a fault parameter estimation approach. IEEE Trans Power Delivery. 2022;37(1):258–67. doi: 10.1109/tpwrd.2021.3056876

[18] Guo MF, Yang NC, Chen WF. Deep-learning-based fault classification using hilbert–huang transform and convolutional neural network in power distribution systems. IEEE Sensors J. 2019;19(16):6905–13. doi: 10.1109/jsen.2019.2913006

[19] Shaikh MS, Lin H, Xie S, Dong X, Lin Y, Shiva CK, et al. An intelligent hybrid grey wolf-particle swarm optimizer for optimization in complex engineering design problem. Sci Rep. 2025;15(1):18313. doi: 10.1038/s41598-025-02154-0 40419534 PMC12106706

[20] Shaikh MS, Raj S, Babu R, Kumar S, Sagrolikar K. A hybrid moth–flame algorithm with particle swarm optimization with application in power transmission and distribution. Decision Anal J. 2023;6:100182. doi: 10.1016/j.dajour.2023.100182

[21] Latif SA, Drieberg M, Sarang S, Abd A, Ahmad R, Stojanovic´ GM. A reinforcement learning based intelligent duty cycle MAC protocol for internet of things. IEEE Access. 2025.

[22] Li X, Ding Q, Sun J-Q. Remaining useful life estimation in prognostics using deep convolution neural networks. Reliabil Eng Syst Saf. 2018;172:1–11. doi: 10.1016/j.ress.2017.11.021

[23] Taye MM. Theoretical understanding of convolutional neural network: concepts, architectures, applications, future directions. Computation. 2023;11(3):52. doi: 10.3390/computation11030052

[24] He K, Zhang X, Ren S, Sun J. Deep residual learning for image recognition. In: Proceedings of the IEEE conference on computer vision and pattern recognition, 2016. 770–8.

[25] Miao Y, Zhang Y, Chen F, Wang Z. Analog circuit incipient fault detection based on attention mechanism and fully convolutional network. IEEE Access. 2024;12:137247–58. doi: 10.1109/access.2024.3403908

